# UV protection properties of workwear fabrics coated with TiO_2_ nanoparticles

**DOI:** 10.3389/fpubh.2022.929095

**Published:** 2022-08-01

**Authors:** Hadiseh Rabiei, Somayeh Farhang Dehghan, Majid Montazer, Shokooh Sadat Khaloo, Aysa Ghasemi Koozekonan

**Affiliations:** ^1^Student Research Committee, Department of Occupational Health and Safety at Work, School of Public Health and Safety, Shahid Beheshti University of Medical Sciences, Tehran, Iran; ^2^Environmental and Occupational Hazards Control Research Center, School of Public Health and Safety, Shahid Beheshti University of Medical Sciences, Tehran, Iran; ^3^Department of Textile Engineering, Amirkabir University of Technology, Functional Fibrous Structures and Environmental Enhancement (FFSEE), Amirkabir Nanotechnoloy Research Institute (ANTRI), Tehran, Iran; ^4^Department of Health, Safety, and Environment (HSE), School of Public Health and Safety, Shahid Beheshti University of Medical Sciences, Tehran, Iran; ^5^Workplace Health Promotion Research Center, Shahid Beheshti University of Medical Sciences, Tehran, Iran; ^6^Department of Health and Safety at Work, School of Public Health and Safety, Shahid Beheshti Medical Sciences, Tehran, Iran

**Keywords:** TiO2, nanoparticles, UPF, *in-situ*, textile

## Abstract

The main purpose of this study was to evaluate the ultraviolet protective factor (UPF) of fabrics coated with TiO2 nanoparticles made using an *in-situ* synthesis method and more accurately assess the intrinsic properties of the textile. The cotton-polyester twill fabric (70–30%) (246.67 g/m2) was coated *in-situ* with TiO2 nanoparticles. *In-situ* coating is conducted in 4 steps; washing the fabrics, preparation of nanoparticles, injecting the nanoparticles into fabrics, and drying the fabric after coating. The scanning electron microscope (SEM) and X-ray diffraction (XRD), FTIR spectrometer, dynamic light scattering (DLS) and UV-Vis spectrophotometer were used to analyse the data of the coating and UPF results. Also, four standards such as ASTM D737, ISIRI 8332, ISIRI 4199, and ISIRI 567 were used for analyzing the intrinsic properties of a textile. The results of SEM, XRD and DLS altogether confirmed the *in-situ* formation of nanoparticles onto textile fibers. Moreover, the UPF value of the uncoated and coated fabrics was 3.67 and 55.82, respectively. It was shown that the *in-situ* deposition of TiO2 nanoparticles on fabric can provide adequate protection against UVR. Also, the results of analyzing the intrinsic properties of the textile showed that there were no significant differences in the intrinsic properties between the coated and uncoated fabrics. Based on the results, it can be concluded that the UV protective properties of workwear fabrics can be improved by coating TiO2 nanoparticles on them without any effect on the cooling effect of perspiration evaporation.

## Introduction

The incidence of skin cancer has been rising at an alarming rate over the past few decades due to overexposure to ultraviolet radiation (1). As a result, UV-induced skin damage has become an urgent health challenge (2–4). Accordingly, different strategies have been introduced to protect the skin against ultraviolet radiation which include using sunscreen, avoiding the sun at its highest intensities, and wearing clothing that effectively covers the skin surface. Outdoor workers such as farm workers, mine workers, bricklayers, construction workers, etc., are exposed to UV radiation, more frequently and for longer periods than people working indoors. The clothing worn by them is considered the most important personal protective equipment, preventing cuts and lacerations as well as contact with chemicals. Their clothing can protect their body from radiant heat and harmful rays (2, 4). Also, welders, furnace operators (steel industry and foundries), and even laboratory technicians (for UV disinfection) are exposed to ultraviolet light (5, 6). Wearing protective clothing is the cheapest and easiest way to limit UV exposure. However, intrinsic properties of textiles such as physical structure and chemical composition, low absorption, and UV–induced vulnerability of textiles can affect their UV-blocking properties. Therefore, the UV-blocking properties of the textile should be optimized. In other words, improvement of the Ultraviolet Protection Factor (UPF) is required to change the physical and chemical properties of textiles (7).

Fabrics generally have a UPF rating of 15–50, allowing only 2.5% of the sun's UV rays to pass through and any fabric that allows <2 percent UV transmission is labeled UPF 50+. Researchers have used different coatings as UV protectors for producing textile fibers. Chemical additives used as UV protectors can become toxic and degraded by sunlight (8, 9). Moreover, the preparation of these protectors often involves several steps as well as the use of hazardous chemicals. Hence, advanced protective coatings should be prepared and modified (10). Given that the properties imparted to textiles using conventional methods often do not have permanent effects and loss of function may occur after repeated washing, while the modifications made to the textile structure using nanotechnology and nanofibers are more stable and can improve the durability of textiles, the use of nanotechnology in the textile industry is increasingly attracting worldwide attention due to the existing interests (11). Nanomaterials such as metal oxide nanoparticles can impart UV blocking properties to textiles (12).

Loading the nanoparticles with UV blocking properties onto nanofibers can have a significant effect on the UV blocking properties of the textile. Nanoparticles are more efficient in blocking UV light than larger particles. Metal oxide nanoparticles such as titanium dioxide (TiO_2_), zinc oxide, selenium dioxide, and aluminum dioxide have been used for this purpose, among which nano-TiO_2_ is one of the best choices due to its cost-effectiveness, and simplicity, low toxicity, and high surface energy (13). Studies have shown that titanium provides UV protection via an absorption mechanism (14). For instance, Attia et al. (15) coated cotton/polyester (65/35%) fabric with TiO_2_ and ZnO nanoparticles (20 nm), and the results showed that UPF in treated fabrics was significantly enhanced and achieved more than six fold. Also, Hasan et al. coated aramid fabrics with silver nanoparticles to assess the antibacterial effects of nanoparticles. Results revealed that the fabrics have demonstrated excellent antibacterial action with more than 99% bacterial reduction efficiency against both Escherichia coli (E. coli) and Staphylococcus aureus (S. aureus) (16). At the end, Mondal showed in a review article that several nanomaterials such as ZnO, TiO_2_, and Carbon nanotube were used in several studies. Also, cotton fabrics were the most used materials to study UPF (17).

Given the vast numbers of outdoor workers and their prolonged exposure to the sun, designing UV-protective textiles is essential since the intrinsic properties of textiles such as physical structure and chemical composition, low absorption, and UV– induced vulnerability of textiles can affect their UV-blocking properties. Therefore, the present study aimed to evaluate the UPF of fabrics coated with TiO_2_ nanoparticles made using an *in-situ* synthesis method and more accurately assess the intrinsic properties of the textile (such as air permeability, abrasion resistance, wrap resistance and surface wetting resistance).

## Materials and methods

### Chemical substances and fabric properties

Chemical substances including Titanium isopropoxide (TTIP) (284.22 g/mol, C1_2_H_2_8O4Ti) (Merck No. 821895), ethanol 99.99%, and sodium hydroxide were purchased from Shimi Parsian Co Tehran, Iran. Also, the cotton-polyester twill fabric (blend ratio: 70/30%) (246.67 g/m^2^) was provided by Yazd Baf Co, Yazd, Iran. To remove the interference effects of dying on the UPF value, the fabric was purchased from the factory before being dyed. The cotton-polyester twill fabric was used since it is the most common workwear fabric in Iran.

### Synthesis of TiO_2_ nanoparticles on the fabric

TiO_2_ nanoparticles were *In-situ* synthesized on cotton fabrics as described as the fabric samples in cuts of 4 × 4 cm were washed with ethanol inside a shaker and then again rinsed in distilled water and dried in an oven at 80°C. Next, a 4.2 mol/L NaOH solution was used for nanoparticle preparation, and the mixture was then subjected to ultrasonic cleaning in an ultrasonic bath (PARSONIC 2600s, Ac 220v/50 Hz) for 15 min. Also, 1 ml of pure ethanol and 4.5 ml of TTIP, and 1 ml of pure ethanol were withdrawn with a 10 ml syringe, respectively. The solution inside the syringe was then added dropwise to the solution containing the fabric in the ultrasonic bath and subjected to the ultrasonic cleaning for 5 h. The fabric samples were then washed three times in distilled water and dried in an oven at 80°C for 2 h.

### Structural properties

The morphological structure of the textiles was investigated using a scanning electron microscope (SEM) and energy-dispersive X-ray spectroscopy system (EXD) (XL30, supplied by Philips Healthcare, the Netherlands) and a Philips PW-1510 diffractometer (PW 1510) before and after direct *in-situ* modification of cotton fabric using TiO_2_ nanoparticles. The samples were placed on the SEM stub and coated with gold. Image Analysis Software was used after x-ray diffraction imaging, morphological analysis, and particle size analysis. An FTIR spectrometer (Tensor 27, Bruker, Ettlingen, Germany) was used to analyze the chemical composition of synthesized TiO_2_ nanoparticles and the amount of metal loaded, and a dynamic light scattering analyzer (Microtrac MRB's NANOTRAC Wave, USA) was used to analyze TiO_2_ nanoparticle size. The energy-dispersive X-ray spectroscopy system, attached to the same microscope, was also used for elemental analysis. The structure of the *In-situ-*modified fabric samples was identified *via* X-ray diffraction (XRD) analysis at room temperature using an X-ray diffractometer (Panalytical X'PERT PRO) with a Cu Kα anode operating at 40 kV and 50 mA and λ = 1.5406). Diffraction data were collected over the 2θ range of 10–50° with a step size of 0.02 ° and a scan speed of 1 s. The use of these instruments as well as how to do it is designed and implemented based on previous studies (18–21).

### UV protection

The UV transmittance spectra (280–400 nm) of the textile were measured at a distance of 5 nm using a spectrophotometer (Varian Cary 100, Australia) (22). The UPF, UV-A (315-400) protection factor, and the UV-B (280-315) protection factor were evaluated using AS/NZ 4399:1996 and equation 1. Three replicated measurements were carried out for each sample and an average value was reported (22). According to the AS/NZ 4399: 1996 standard, the spectral intensity of radiation proportional to that of the solar spectrum (S_λ_) in the spectrophotometer was proportional to any wavelength from 290 nm (4–10 × 767/0 W.m^−2^.nm^−1^) to 400 nm (180/1 W.m^−2^.nm^−1^).

Equation 1 (22).


(1) $$UPF=\frac{\int_{280}^{400}E_{\text{λ}}S_{\text{λ}}d_{\text{λ}}}{\int_{280}^{400}E_{\text{λ}}S_{\text{λ}}d_{\text{λ}}T_{\text{λ}}}$$

Where:

E_λ_: Relative efficiency of the radiation source.
S_λ_; Initial spectrum of the radiation source (W.m-2. nm-1).
d_λ_: Bandwidth (nm).
T_λ_: The amount of light transmitted.

### Intrinsic properties of the manufactured textiles

The intrinsic properties of fabrics contribute to their performance, resistance, and heat transfer, so the effect of nanoparticles, applied as a coating, on these properties should be investigated. The properties examined in this study include:

1- Air permeability: the rate of airflow passed through the pores. This property, which indicates the fabric's resistance to sweating-induced evaporative cooling, was tested *via* ASTM D737 method using the TF164E Air Permeability Apparatus (23).

2- Abrasion resistance: the ability to resist wearing and indicates the cut resistance. This property was estimated using ISIRI 8332 standard (24).

3- Wrap resistance: resistance to tensile force and indicates fabric's strength against tearing. This property was evaluated using ISIRI 4199 (25).

4- Surface wetting resistance: the penetration of water through the fabric or the resistance to wetting. It was measured using ISIRI 567 (26).

## Results and discussion

### Properties of the fabrics coated with TiO_2_ nanoparticles

**Figure 1:**
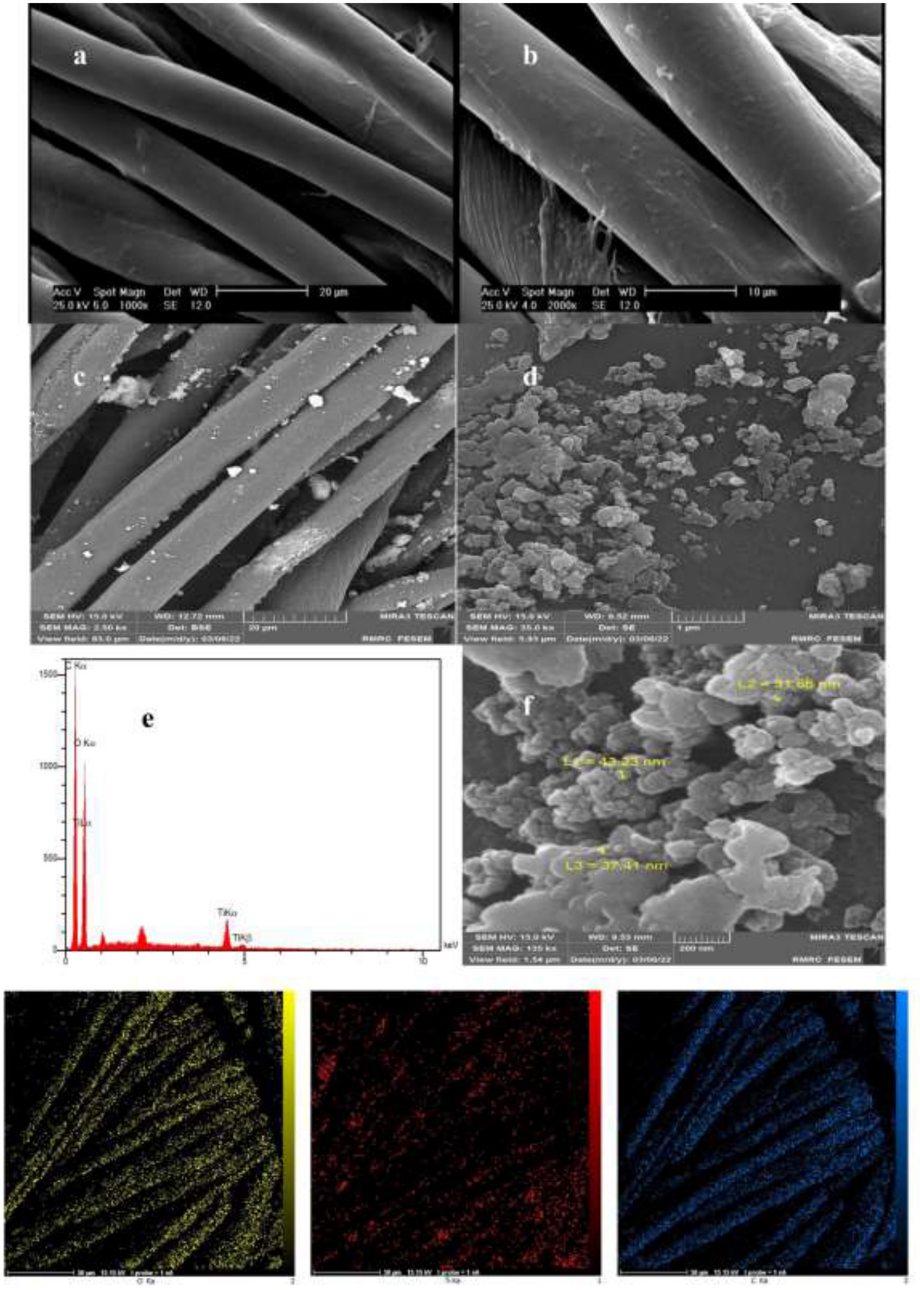
EDX and SEM analysis of TiO_2_ nanoparticles coating: **(a,b)** Imaging of the uncoated fabric with image sizes of 20 and 10 μm. **(c,d,f)** Imaging of TiO_2_ -coated fabric with image sizes of 20, 1 μm, and 500 nm. **(e)** EDX spectra of the agents coated onto the fabric surface.

**Figure 2:**
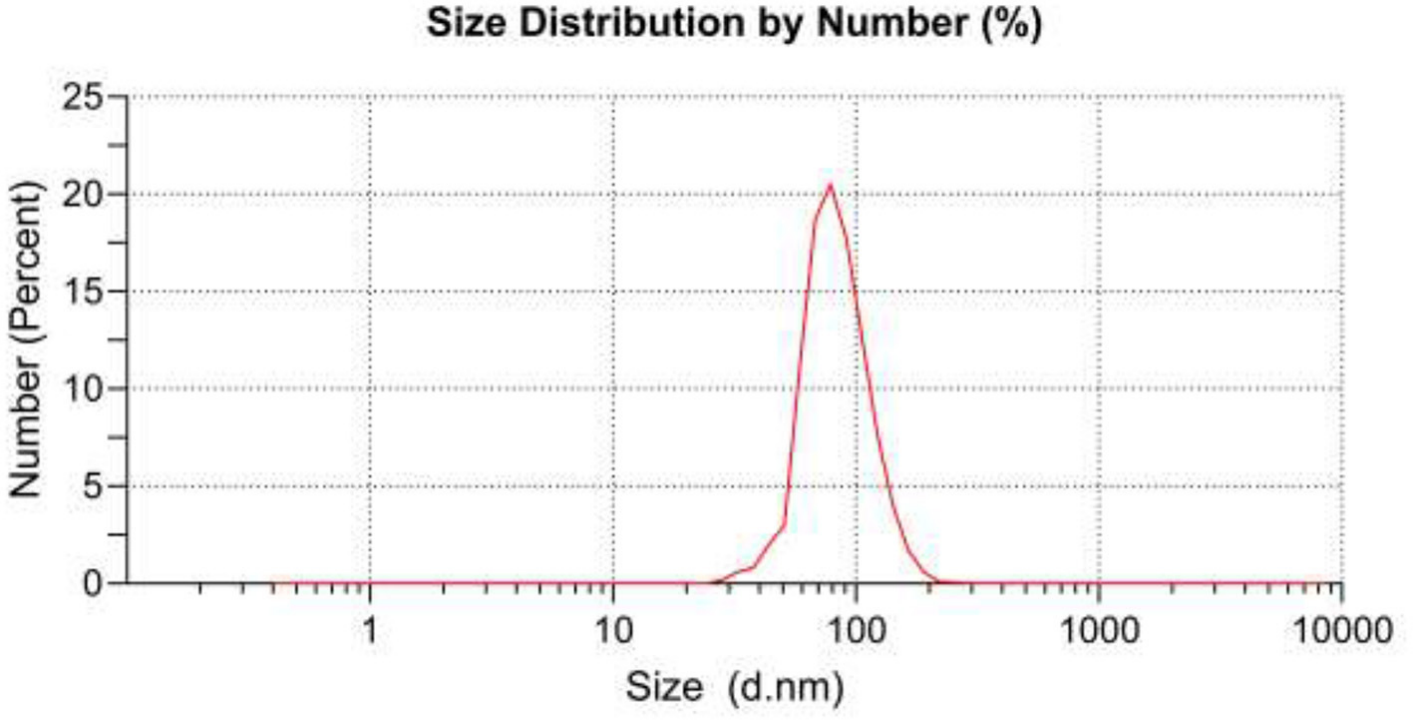
Size distribution of TiO_2_ nanoparticles obtained by DLS.

Coating thickness was determined by gravimetric analysis. The results showed that the weight of the fabric was 0.4297 g before coating and 0.4745 g after coating, so 10.42% of TiO_2_ nanoparticles had been coated on the surface of the fabric. Micrographs and EDX analysis as well as SEM images confirmed the *in-situ* formation of nanoparticles onto the textile fibers (Figure 1). Ti signals observed in EDX analysis patterns of the fabric modified with TiO_2_ nanoparticles were recorded. The diameter of TiO_2_ nanoparticles on the textile fibers was measured using the DLS method. The nanoparticle average diameter determined by DLS was found to be 98.15 nm (Figure 2). Dense TiO_2_ nanoparticles were observed on the fabric, indicating the effect of the chemical composition of the fabric on the nature of the prepared TiO_2_ nanoparticles (27, 28). The EDX spectra revealed the presence of C, O, and Ti on the surface of cotton fabrics.

**Figure 3:**
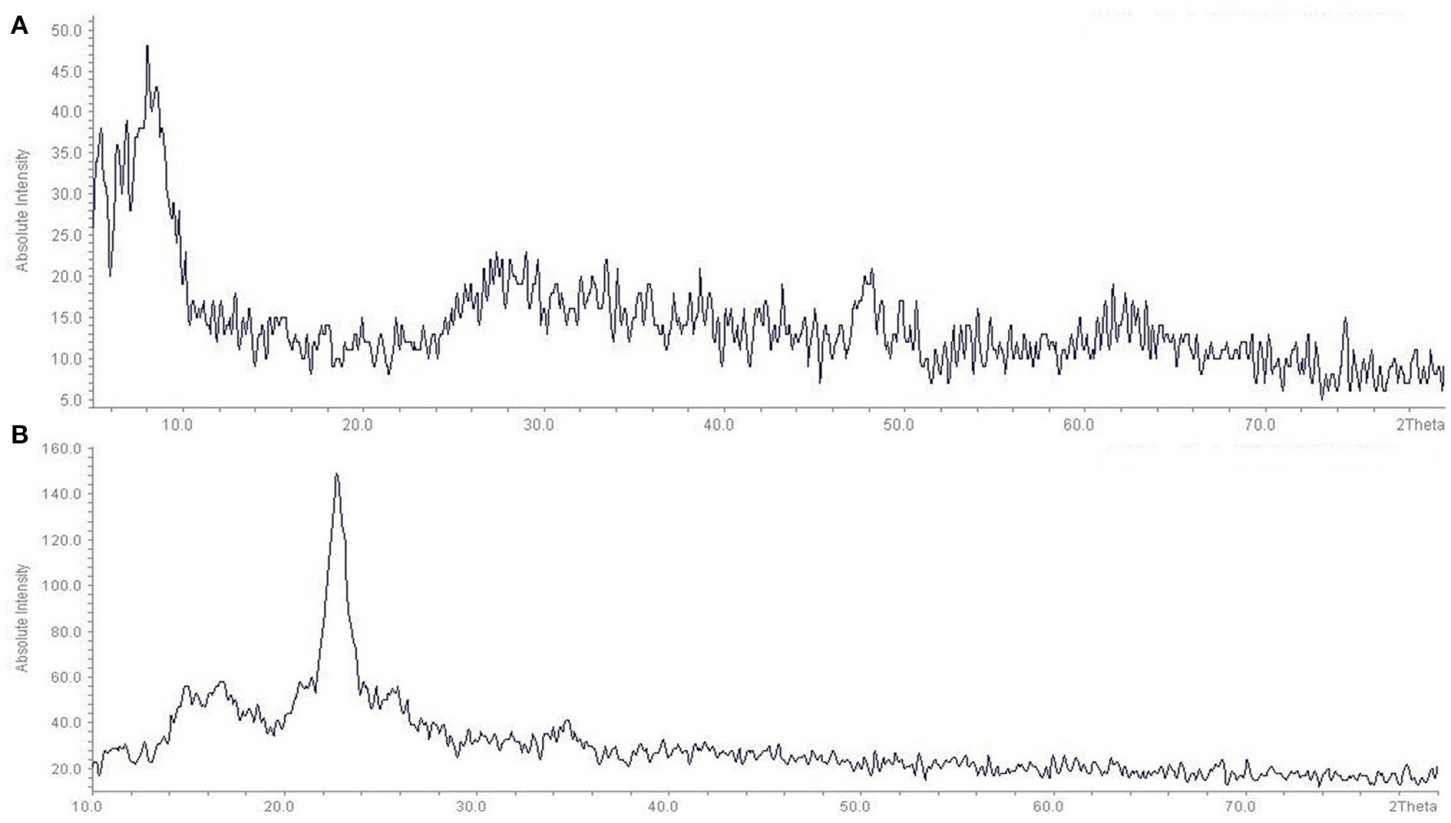
XRD patterns of pure TiO_2_ Nanopowder and fabric coated with TiO_2_ nanoparticles: **(A)** Pure TiO_2_ Nanopowder, **(B)** fabric coated with TiO_2_ nanoparticles.

The XRD analysis confirmed the *in-situ* synthesis of the TiO_2_ on the fabric surface (Figure 3). As can be seen, the diffraction peaks were located at 5.45, 6.90, 8.08, and 29.37°. Also, consistent with previous studies, after surface modification, some 2θ peaks (base peaks) of the TiO_2_ nanoparticles were located at 16.74, 22.75, and 34.70° according to the TiO_2_ nanoparticles (29). The results of the SEM, XRD, FTIR, DLS of the present study are similar to the results of other studies (30–32).

**Figure 4:**
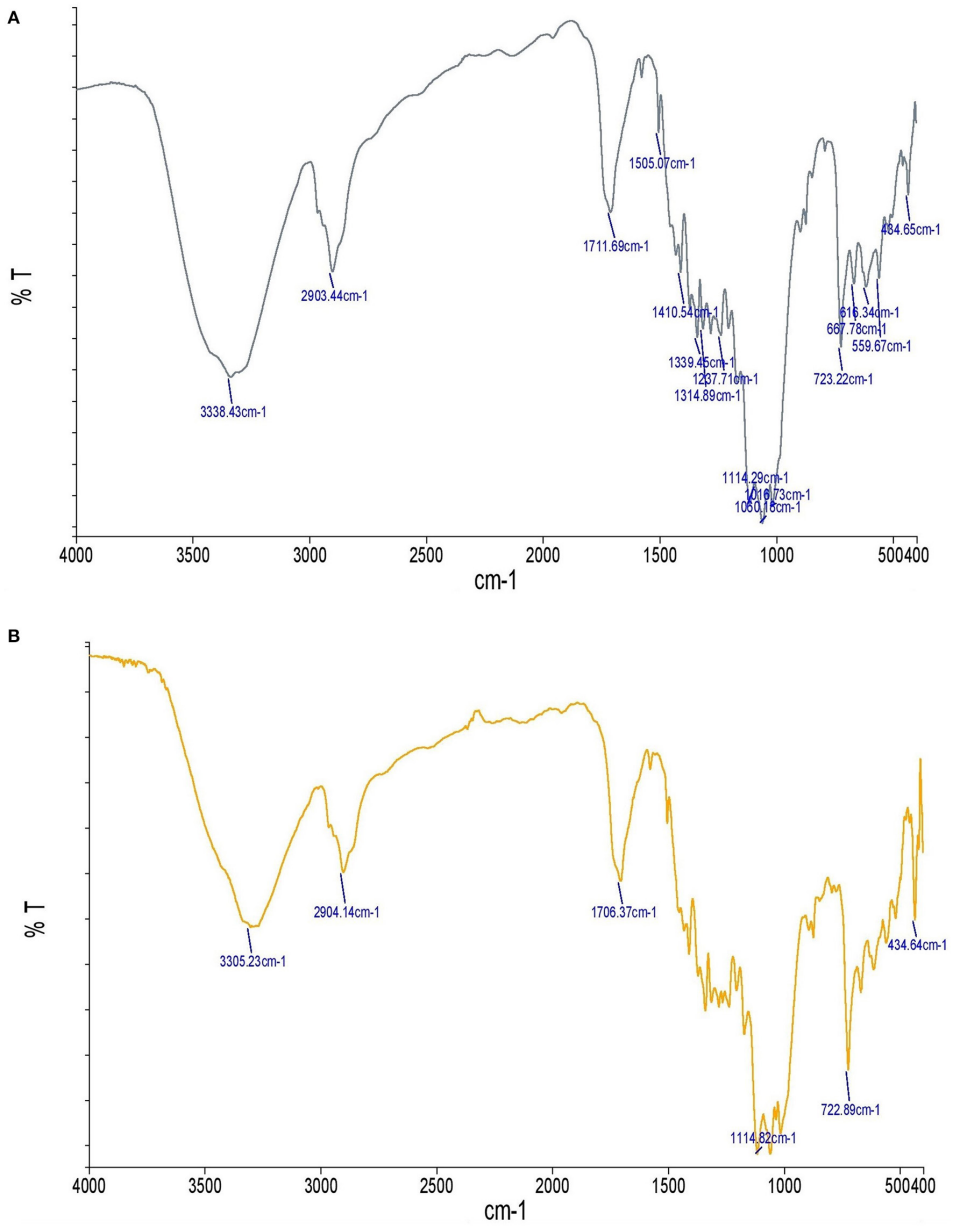
FTIR spectra of uncoated fabric compared with the fabric coated with TiO_2_ nanoparticles: **(A)** Uncoated fabric, **(B)** fabric coated with TiO_2_ nanoparticles.

The chemical composition of the fabric was investigated using FTIR before and after the *in-situ* formation of the TiO_2_ nanoparticles (Figure 4). Note the broad peak at 3,305 cm-1 due to the O–H stretch. The broad peak around 2,904 cm-1 also resulted from the C-H stretch. Despite the CH groups of cellulose, the symmetric and asymmetric stretching peaks were not separated as sharp peaks. The peak near 1,706 cm-1 can be attributed to the absorption of water molecules. The broad absorption bands at 434 cm-1 and 722 cm-1 were caused by the bending vibrations of the Ti-O and -O-Ti-O groups, and the peak observed at 1,114 cm-1 was due to the bending vibration of Ti-O-Ti. The vibration bands observed at 1,300–4,000 cm-1 can be attributed to the chemical and/or physical co-adsorption of the H_2_O and CO_2_ molecules. Therefore, it can be concluded that the fabrics were well coated with the TiO_2_ nanoparticles. These results are consistent with other studies on TiO_2_ nanoparticles (29, 33–37).

### UPF values

**Figure 5:**
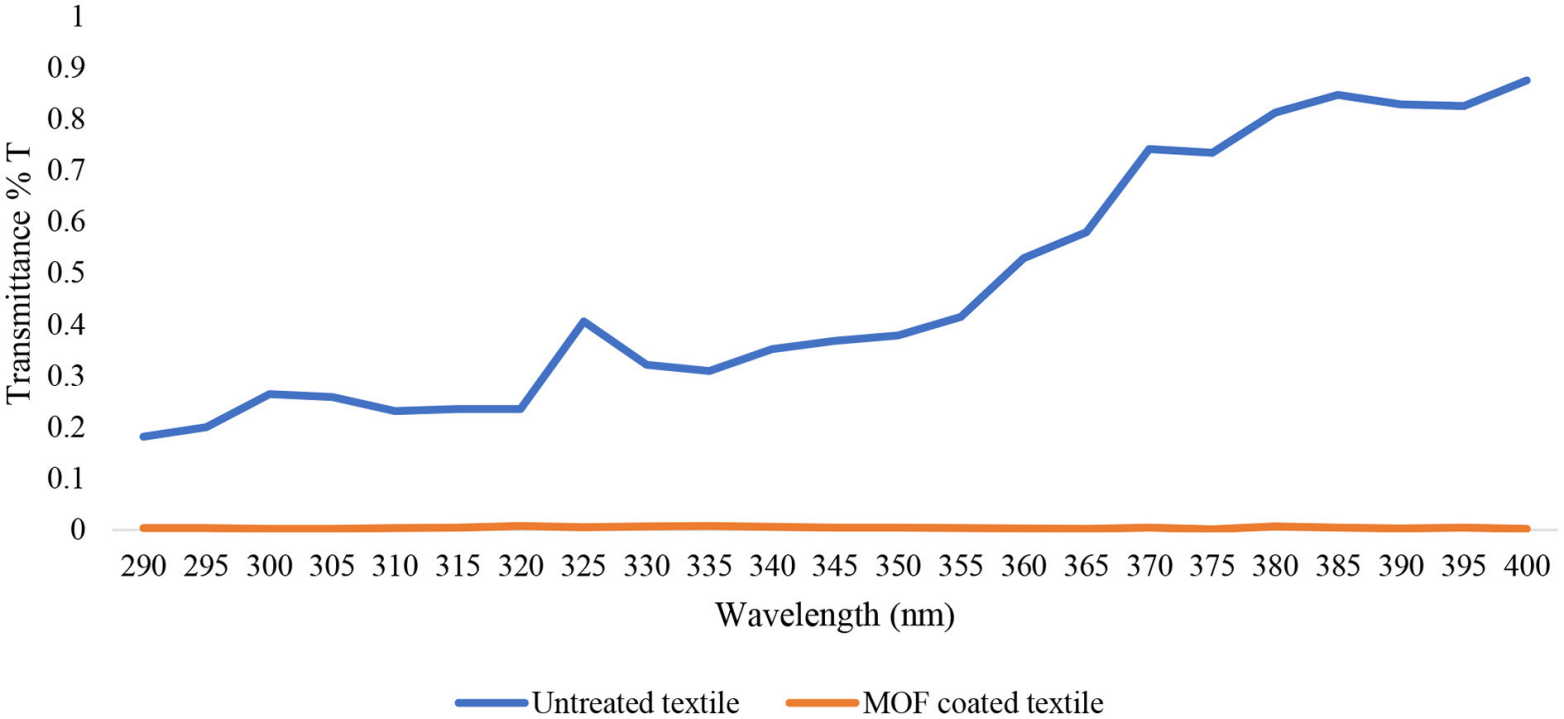
UVR transmittance property of uncoated fabrics and fabrics coated with TiO_2_ nanoparticles according to AS/NZS 4399 Sun Protective Clothing standard.

The UPF of uncoated and coated fabric by measuring the transfer rate (T) for UVR and its spectral results is shown in Figure 5. According to the results, the UPF value of the uncoated fabrics calculated *via* Equation 1 was 3.67 and the UPF value of fabrics coated with TiO_2_ nanoparticles was 55.82, indicating the strong UV blocking ability of the coated fabrics. It was confirmed that the *in-situ* deposition of TiO_2_ nanoparticles on fabric can provide adequate protection against UVR.

According to the Australian/New Zealand Standard and the British standard (22), uncoated fabrics with UPF < 20 have insufficient UV protection. After direct *in-situ* modification, the fabrics reached UPF > 50 and could be classified in the excellent UV protection category according to the mentioned standard. Other studies have also shown that TiO_2_ nanoparticles coated on fabric can provide adequate protection against UVR (38, 39). Moreover, previous studies have revealed the enhancement of UV absorption in the TiO_2_ nanoparticles formed on cotton fabrics. The cotton fabrics loaded with dense layers of the TiO_2_ nanoparticles exhibited greater UPF values after a longer deposition period (40). Alebeid et al. also showed that the TiO_2_ nanoparticles can block UV more efficiently compared with dyes, implying that dye is not a reliable indicator of the UV protection provided by dyed fabrics (41–43).

Some studies have shown lower UPF values and this difference can be due to fiber type. For example, Kathirvelu et al. found that 100% cotton fabrics coated with the TiO_2_ had UPF values lower than the adopted standard and it cannot be classified in the excellent “UV protection category. However, cotton and polyester blended fabrics treated with the TiO_2_ nanoparticles exhibited higher UPF values and woven fabrics made of polyester-cotton blend yarns showed better UV absorption properties compared to other fabrics (44). Consistent with other studies, the present study indicated that coating fabrics with TiO_2_ nanoparticles can significantly increase UPF values and improve anti-ultraviolet performance. The difference in the anti-ultraviolet performance can be attributed to different concentrations of the TiO_2_ nanoparticles and increasing the concentration of TiO_2_ nanoparticles can increase the UV protection capacity of the fabric (45). In addition, according to studies, the increase in UPF values can be attributed to the natural UV absorption properties of TiO_2_ and can be explained by the band theory of solids. The TiO_2_ is a semiconductor with a wide bandgap (3.2 eV) between the low energy valance band and the high energy conduction band. When the TiO2 is activated with light waves of energy greater than its bandgap, the electrons will absorb UV light due to its wide bandgap; that is why the TiO_2_ can protect against ultraviolet radiation (46).

Many studies have investigated the UV-blocking capacity of TiO_2_ nanoparticles and have shown that the *in-situ* synthesis of TiO_2_ nanoparticles on the fabric surface can lead to UV protection capacity, confirming that *in-situ* deposition of TiO_2_ nanoparticles on fabric can provide good to excellent UV protection (28, 29).

### Structural properties of the fabric

The present study compared the fabric coated with the TiO_2_ nanoparticle and the uncoated fabric to investigate the effect of coating on the intrinsic properties of the fabric, including wrap resistance, air permeability, abrasion resistance, and surface wetting resistance. Wrap resistance and abrasion resistance indicate the ability of fabric in various conditions and environments. Also, air permeability and surface wetting resistance refer to the rate of airflow and moisture transfer through the fabric, respectively. This can affect the sweat absorption and comfort properties of the fabric. The results showed no significant difference in the intrinsic properties between the coated and uncoated fabrics, implying that coating the fabric with the TiO_2_ nanoparticles had no effect on the intrinsic properties of the fabric and caused no reduction in its resistance, air permeability, and the cooling effect of perspiration evaporation (Table 1). This lack of difference shows that coating the fabrics with the TiO_2_ nanoparticles did not affect their intrinsic properties and the heat and air transfer capacities of the modified fabric were similar to those of the uncoated fabric. Also, the modified fabric had sufficient resistance to withstand work environments. Few studies have investigated the intrinsic properties of the fabric after coating it with the TiO_2_ nanoparticles and the majority of studies conducted in this field aimed to make fabrics waterproof. No study has been conducted to design workwear fabrics with high UPF values without imposing changes to their intrinsic properties. However, previous studies have also shown that hydrophilicity is also an important property that refers to absorbing moisture vapor and can add to the comfort properties of fabric (47, 48).

**Table 1:** Comparison of uncoated and coated textile with TiO_2_ nanoparticle in terms of intrinsic properties of the textile.

**Textiles**	**Intrinsic properties of the textile**							
	**Warp resistance N), Mean (SD)**	***P*** **-value** ^*^	**Air Permeability (ml/cm** ^2^ **/s), Mean (SD)**	***P-*** **value**	**Abrasion resistance (kg), Mean (SD)**	***P*** **-value**	**Surface wetting resistance (g), Mean (SD)**	***P*** **-value**
Uncoated	16.19 (3.96)	0.10	21 (1.58)	0.073	204 (1.16)	0.317	0.47 (0.01)	0.317
Coated with TiO_2_	16.32 (1.62)		18.80 (1.64)		501.66 (1.15)		0.67 (0.01)	

* *Mann-Whitney Test*.

## Conclusion

The present study aimed to evaluate the UPF (ultraviolet protective factor) of fabrics coated with the TiO_2_ nanoparticles made using an *in-situ* synthesis method and more accurately assess the intrinsic properties of the textile. The results showed that the textile coated with the TiO_2_ nanoparticle had greater UPF values than the uncoated textile and also the intrinsic properties of the coated fabric did not change significantly. Based on the results, it can be concluded that the UV protective properties of workwear fabrics can be improved by coating the TiO_2_ nanoparticles on them without any effect on the cooling effect of perspiration evaporation. However, the production of workwear fabrics requires further research. Future studies can evaluate the cytotoxicity and antibacterial properties of the TiO_2_ nanoparticles to ensure that the TiO_2_ coating of fabrics has no adverse effects on human skin.

## Data availability statement

The original contributions presented in the study are included in the article/supplementary material, further inquiries can be directed to the corresponding author.

## Ethics statement

Ethical approval for this study was obtained from School of Public Health and Neuroscience Research Center, Shahid Beheshti University of Medical Sciences (IR.SBMU.PHNS.REC.1400.045).

## Author contributions

SF was the leader of study and edited the final manuscript. HR and AK gathered data and performed laboratory experiments and were a major contributor in writing the manuscript. MM analyzed laboratory experiments about fabrics and was a major contributor in writing the manuscript. SK analyzed nanomaterials laboratory experiments and was a major contributor in writing the manuscript. All authors read and approved the final manuscript. All authors contributed to the article and approved the submitted version.

## Funding

This study was part of the research projects supported by Shahid Beheshti University of Medical Sciences (Grant No. 28591).

## Conflict of interest

The authors declare that the research was conducted in the absence of any commercial or financial relationships that could be construed as a potential conflict of interest.

## Publisher's note

All claims expressed in this article are solely those of the authors and do not necessarily represent those of their affiliated organizations, or those of the publisher, the editors and the reviewers. Any product that may be evaluated in this article, or claim that may be made by its manufacturer, is not guaranteed or endorsed by the publisher.
